# Accurate assessment of LV function using the first automated 2D-border detection algorithm for small animals - evaluation and application to models of LV dysfunction

**DOI:** 10.1186/s12947-019-0156-0

**Published:** 2019-04-22

**Authors:** Jana Grune, Daniel Ritter, Kristin Kräker, Kathleen Pappritz, Niklas Beyhoff, Till Schütte, Christiane Ott, Cathleen John, Sophie van Linthout, Carsten Tschöpe, Ralf Dechend, Dominik N. Muller, Nadine Haase, Tilman Grune, Ulrich Kintscher, Wolfgang M. Kuebler

**Affiliations:** 1 Institute of Physiology, Charité-Universitätsmedizin Berlin, Charitéplatz 1, 10117 Berlin, Germany; 2 German Centre for Cardiovascular Research (DZHK), partner site Berlin, 10117 Berlin, Germany; 3 Institute of Pharmacology, Center for Cardiovascular Research, Charité - Universitaetsmedizin Berlin, Hessische Straße 3-4, 10115 Berlin, Germany; 4 Experimental and Clinical Research Center, a joint cooperation of Max-Delbrück Center for Molecular Medicine and Charité - Universitätsmedizin Berlin, 13125 Berlin, Germany; 5 Berlin Institute of Health (BIH), Berlin, Germany; 6 Max-Delbrück Center for Molecular Medicine, 13125 Berlin, Germany; 7 Berlin – Brandenburger Center for Regenerative Therapies (BCRT), Charité – Universitätsmedizin Berlin, Campus Virchow Klinikum (CVK), Berlin, Germany; 8 Department of Molecular Toxicology, German Institute of Human Nutrition Potsdam-Rehbruecke (DIfE), 14558 Nuthetal, Germany; 9 Department of Internal Medicine and Cardiology, Charité – Universitätsmedizin Berlin, Campus Virchow Klinikum (CVK), Berlin, Germany; 10 HELIOS-Klinikum, Berlin, Germany; 11 German Center for Diabetes Research (DZD), 85764 Muenchen-Neuherberg, Germany; 12 Charité – Universitätsmedizin Berlin, Freie Universität Berlin, Humboldt-Universität zu Berlin and BIH, Berlin, Germany; 13 Departments of Surgery and Physiology, University of Toronto and Keenan Research Centre for Biomedical Science of St. Michael’s, Toronto, Canada

**Keywords:** Echocardiography, Automated border detection, LV systolic function, Small animals

## Abstract

Echocardiography is the most commonly applied technique for non-invasive assessment of cardiac function in small animals. Manual tracing of endocardial borders is time consuming and varies with operator experience. Therefore, we aimed to evaluate a novel automated two-dimensional software algorithm (Auto2DE) for small animals and compare it to the standard use of manual 2D-echocardiographic assessment (2DE). We hypothesized that novel Auto2DE will provide rapid and robust data sets, which are in agreement with manually assessed data of animals.

2DE and Auto2DE were carried out using a high-resolution imaging-system for small animals. First, validation cohorts of mouse and rat cine loops were used to compare Auto2DE against 2DE. These data were stratified for image quality by a blinded expert in small animal imaging. Second, we evaluated 2DE and Auto2DE in four mouse models and four rat models with different cardiac pathologies.

Automated assessment of LV function by 2DE was faster than conventional 2DE analysis and independent of operator experience levels. The accuracy of Auto2DE-assessed data in healthy mice was dependent on cine loop quality, with excellent agreement between Auto2DE and 2DE in cine loops with adequate quality. Auto2DE allowed for valid detection of impaired cardiac function in animal models with pronounced cardiac phenotypes, but yielded poor performance in diabetic animal models independent of image quality.

Auto2DE represents a novel automated analysis tool for rapid assessment of LV function, which is suitable for data acquisition in studies with good and very good echocardiographic image quality, but presents systematic problems in specific pathologies.

**Electronic supplementary material:**

The online version of this article (10.1186/s12947-019-0156-0) contains supplementary material, which is available to authorized users.

## Introduction

Echocardiography is the standard of use for the assessment of left ventricular (LV) function in clinical routine and basic research [1, 2]. Despite recommendations for the use of three-dimensional echocardiography (3DE) [3], two-dimensional echocardiography (2DE) still presents a relatively inexpensive, straightforward and time saving method for the non-invasive assessment of LV function as compared to 3DE and gold-standard cardiac magnetic resonance imaging (CMR) and is therefore often the method of choice [1, 2]. Typically, 2DE analysis in small animals is based on the traditional monoplane Simpson’s method of discs where endocardial border regions are traced in a single image plane, usually the maximum dimension of the LV [4]. The procedure of endocardial border tracing has to be repeated in end-systolic and end-diastolic frames to allow the calculation of e.g. LV ejection fraction (EF), a key parameter for diagnosis, management and treatment of cardiac pathologies [2, 3, 5]. Despite the seeming simplicity of 2DE analysis, large inter-individual variabilities have been reported for this analysis in both humans and small animals [6–8]. Moreover, quantitative analysis of 2DE is highly dependent on operator experience and analysis of imaging data requires a significant investment of time, especially for basic research studies with large n-numbers [9].

**Fig. 1:**
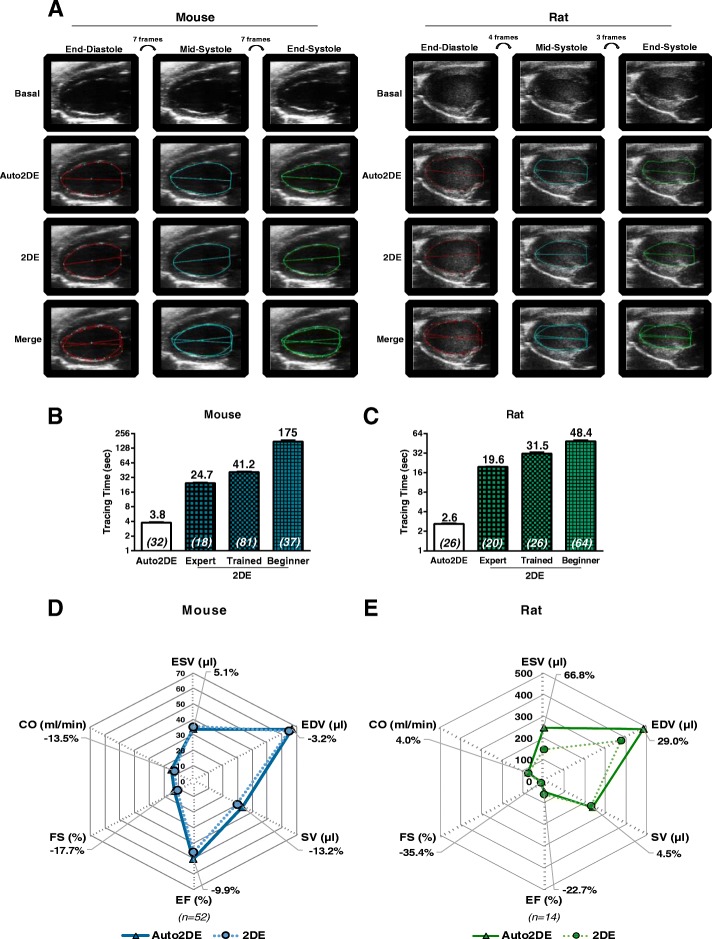
Automated assessment of LV function in healthy mice and rats. **a** Exemplary cine loops +/− tracings by conventional 2DE and novel Auto2DE in healthy mice and rats. **b** Mean tracing time for mouse and **c** rat cine loops by operators of distinct experience levels. Numbers in brackets indicate numbers of tracings. **d** Average Auto2DE-assessed LV function parameters in comparison to standard 2DE-assessed data sets in cohorts of 52 mouse cine loops and **e** 14 rat cine loops. Bold printed numbers indicate the percentage mean difference between Auto2DE and 2DE. Numbers in brackets indicate n-numbers

Required operator experience and time consumption, the major drawbacks of conventional 2DE analysis, might be overcome with the use of novel automated software tools. Automated software approaches have been used earlier in clinical research and demonstrated rapid and reproducible assessment of LV function with very good agreements between automated software tools and manually assessed results [2, 9–12]. Advancements in ultrasound technology recently also paved the way for the first automated 2D-border detection algorithm (Auto2DE, FUJIFILM VisualSonics, Toronto, Ontario, Canada) for the assessment of LV systolic function in small animals. Auto2DE represents a clinically proven edge-matching algorithm trained with a library of over 200 expertly curated LV analysis traces. Cine loops of choice are automatically tested against this library, resulting in the generation of a potentially user-modifiable tracing of LV endocardial borders across an automatically selected series of frames (Fig. 1a). While the advantages of such a novel automated algorithm for basic research cannot be overstressed, its usefulness critically depends on its ability to yield valid and reproducible data and its ability for rapid data analysis independent of observer experience.

To our knowledge, this automated software approach for small animals has not been evaluated to date. In the present study we therefore utilized novel Auto2DE for the assessment of LV function and compared obtained values with manually 2DE-assessed data in healthy and diseased animals. We hypothesized that Auto2DE is able to rapidly provide accurate data, which correlate well with 2DE values. We also speculated that the strength of this relationship is likely dependent on image quality, which may limit Auto2DE performance in severe cardiac pathologies.

## Material and methods

All animal procedures were performed in accordance with the German Law on Protection of Animals and according to the European legislation (Directive 2010/63/EU) and were approved by the local authorities (Landesamt für Gesundheit und Soziales, Berlin, Germany). Animals used in this study served as controls in ongoing projects or were partly already described in recent publications (transverse aortic constriction (TAC), Isoproterenol-induced subendocardial fibrosis (Fib) and a double-transgenic rat model of heart failure with preserved ejection fraction (HFpEF; dTGR cohorts)) [7, 13, 14].

### Validation cohorts in mice and rats

Animals were kept under identical housing conditions (12 h light/dark cycle, standard diet ad libitum, 21 °C room temperature) prior to echocardiographic assessment.

Echocardiographic data sets from 13 healthy male control mice (strain: *Ncor1*^*tm1Anh*^/J (the Jackson Laboratory, JAX stock #017632) were used as murine validation cohort. Each individual mouse was imaged fourfold at the age of 8, 12, 15, and 18 weeks. From the resulting 52 echocardiographic data sets 2 were rated with image quality Q4 (for details see Assessment of image quality-section below) and therefore excluded from further analysis, the remaining 50 data sets entered further analysis.

For the rat validation cohort, a set of 14 echocardiographic cine loops from 14 healthy rats (strain: sprague dawley rat, Max-Delbrück Center; 7 and 18 weeks of age) was used.

### Cardiovascular disease models

For induction of type I diabetes mellitus (DM), male *Ncor1*^*tm1Anh*^/J mice (8–9 weeks) were injected with streptozotocin (50 mg/kg/d STZ, *n* = 12) or vehicle (Ctrl, *n* = 14) for 5 consecutive days. Animals were starved prior to STZ- or vehicle injections for 4 h. Blood glucose levels were determined with a Contour XT glucose meter (Bayer Health Care; Leverkusen, Germany). Echocardiography and blood glucose measurements were performed 12 weeks after DM induction.

As model of type II DM, homozygous BKS.Cg-m+/+Lepr^db^/BomTac (db^+^/db^+^, *n* = 15) mice (Taconic; Skensved, Denmark) carrying a leptin receptor mutation were used. Heterozygous (db^+^/db^−^) littermates served as controls (*n* = 7). At 20 weeks of age, echocardiography was performed and blood glucose levels were determined by Accu-Chek Aviva® (Roche Diabetes Care Deutschland GmbH; Mannheim, Germany) after 4 h fasting. With respect to Table 2, values for heart weight (HW), body weight (BW) and Heart-weight-to-body-weight-ratios (HW/BW) were reported at 24 weeks of age.

Subendocardial fibrosis was modeled as described previously by us [13, 15]. In brief, 6–8 weeks old male 129/Sv mice (*n* = 9, Janvier Labs; LeGenest-Saint-Isle, France) were injected s.c. with isoproterenol (Fib, 25 mg/kg/d; dissolved in saline) or vehicle (saline, *n* = 10) for four consecutive days. Echocardiographic examinations were performed 12–13 days after the final treatment, and final necropsy was performed on day 14.

Mechanical loading as a model of LV failure was induced in male C57BL/6 J mice (8–9 weeks, *n* = 9) by transverse aortic constriction (TAC) as previously reported by us [7, 16]. SHAM-operated animals without banding served as controls (*n* = 7). Echocardiography was performed 10 weeks after TAC or SHAM-surgery, and final necropsy was performed one day later.

Generation of the inducible transgenic rat model for DM was described previously by us [17]. In brief, male tetO-shIR rats (TetO, 18 weeks) received 2 mg/kg/d doxycycline (DOX) via drinking water until blood glucose levels reached 300–400 mg/dl (*n* = 10). Baseline measurements of tetO-shIR served as controls (*n* = 10). Afterwards, we administered 0.5 mg/kg/d DOX over an entire period of 8 weeks. Echocardiographic image acquisition was carried out 8 weeks after initial DM induction. Same experimental protocol was performed using age-matched hypertensive Ren-2 transgenic TGR (mREN2)27 rats (mRen) [18] and a cross breeding of them with tetO-shIR (TetO/mRen), suffering from the metabolic syndrome.

The double-transgenic rat (dTGR) model of experimental HFpEF, a cross-breed of TGR(hRen)L10 J (female breeder) and TGR(hAogen)L1623 (male breeder) (dTGR, 10% transcutol, 20% cremophor, 70% water *n* = 8) and nontransgenic SD control rats (*n* = 5) were treated by oral gavage once daily [14]. Treatment was started at the age of 4 weeks until the end of the study. Echocardiographic image acquisition was performed directly before euthanasia at week 6.5.

### Echocardiographic image acquisition

Echocardiography was carried out as recently described by us [7, 13, 14]. Briefly, we used ultra-high frequency linear array transducers (mice: MX400 18–38 MHz, center transmit: 30 MHz, axial resolution: 50 μm; rats: MX250 13–24 MHz, center transmit: 21 MHz, axial resolution: 75 μm) coupled to a Vevo® 3100 (mice) or a Vevo® 2100 (rats) high-resolution Imaging System (all FUJIFILM VisualSonics; Toronto, Ontario, Canada). Animals were anesthetized with 3% isoflurane (Baxter International, Deerfield, Illinois, USA) and fixed in supine position on a heatpad at 37 °C (FUJIFILM VisualSonics, Toronto, Ontario, Canada). Isoflurane concentrations were further reduced to a minimum of 1–2% to achieve constant and comparable heart rates during image acquisition. B-Mode cine loops were generated visualizing the maximum dimension of the LV from apex to base in a parasternal long axis view. All acquired images were digitally stored in raw format (DICOM) for further offline-analyses.

### Analysis with conventional 2D-echocardiography

2DE analysis was performed using the semi-automated *LVtrace*-Tool of the dedicated software package *VevoLAB* Version 3.0 (FUJIFILM VisualSonics; Toronto, Ontario, Canada), which is based on the monoplane Simpson’s method of discs. Semi-automated 2DE tracings were generated by manual selection of end-diastolic and end-systolic dimension of the LV by each observer. To avoid variations due to sinus cycle length and respiration artifacts the observer reviewed several cardiac cycles of a cine loop and selected a suitable cycle prior to the tracing. 2DE tracings followed the endocardial border regions, covering the whole LV from apex to base. All B-Mode cine loops were traced twice with gold standard 2DE to account for interbeat variability, and resulting values were averaged for the final 2DE data set. Details regarding the assessment of tracing time can be found in the Additional file 1.

### Automated assessment of LV function

The same set of B-Mode cine loops was used for conventional 2DE and novel Auto2DE analysis (Vevo Lab Version 3.1.0 (Build 13,029), FUJIFILM VisualSonics; Toronto, Ontario, Canada). 2DE tracings were carried out prior to Auto2DE analysis, ensuring adequate blinding of the manual evaluation. 2DE-derived tracings and data were not visible to the operator performing Auto2DE. For analysis of Auto2DE, B-Mode cine loops of the LV were manually navigated to an R-wave of the simultaneously recorded electrocardiogram by visually running through the cine loop. If this procedure was hampered by pathophysiologic alterations of the electrocardiogram, the maximum dimension of the LV was manually visualized by the observer. The automated tracing was realized by using the *AutoLV*-tool of the *VevoLab* software (further referred to as Auto2DE). The chosen image is automatically tested against a library to produce a tracing of the LV endocardial borders across a series of frames. The tool was developed by adapting the clinically accepted modified Simpson’s monoplane method of disks approach for LV analysis. Clicking the *AutoLV*-button in the measurement panel automatically produces a tracing of the LV endocardial border on each frame from the starting R-wave (diastole) forward to the next P-wave (systole). Hence, automated analysis of the same frame twice, would result in identical values of cardiac function parameters, guaranteeing observer-independence. To account for interbeat variability, all B-Mode cine loops were traced twice with Auto2DE, choosing two different frames for analysis. The resulting values were subsequently averaged in the final Auto2DE data set.

### Assessment of image quality

Image quality was classified based on visibility of segments and endocardial borders as Q1 (good), Q2 (fair), Q3 (poor), or Q4 (insufficient). Cine loops with Q4 were excluded from all further analysis. The classification of image quality was graded by an expert in small animal echocardiography as follows: Q1 = clearly delineated endocardial border regions and all segments clearly visible, no apex foreshortening; Q2 = slightly diffuse endocardial visualization, occasional minor rib shadows or artifacts in apex or base regions; Q3 = diffuse endocardial border delineation combined with moderate artifacts or rib shadows or apex foreshortening; Q4 = insufficient endocardial visualization or one or more segments covered by artifacts or rib shadows.

### Statistics

All analyses were done using Prism 7 software (GraphPad Software, La Jolla, CA). Results are shown as mean ± standard error of the mean (SEM) with/without individual scatters. Statistical analyses were performed using one-way-ANOVA for multiple comparisons followed by Uncorrected Fisher’s LSD posttest. Correlation of variables of diseased cohorts was tested using Pearson’s correlation coefficient (r). Correlation between methods was defined as follows: r > .8 very good, r > .6 good, r > .4 moderate and r < .4 poor. A *p*-value of <.05 was assumed as statistically significant. 2DE and Auto2DE were compared by Bland-Altman plots and results expressed as bias and limits of agreement (LOA). Post-hoc power analysis was computed based on effect size, sample size and type I error α, using the G*Power 3.1.9.4 freeware tool (Heinrich Heine University, Duesseldorf, Germany).

## Results

### Automated assessment of LV function is faster than conventional 2DE analysis

One of the major benefits to be expected from automated software algorithms is time effectiveness. To this end, we compared the average time required for LV tracings with conventional 2DE from observers with distinct experience levels and novel Auto2DE in mice and rats (Fig. 1b, c). Tracing times for rat cine loops were consistently shorter than tracing times in mice, independent of experience levels. For both rats and mice, observer experience level had expectedly a strong effect on tracing time, with less trained observers requiring longer tracing times. Direct comparison of both software tools demonstrated that mean tracing times of novel Auto2DE were 6.5–7.5 fold faster than the fastest observer and 18–46 fold faster than the slowest observers for each species. To assess accuracy of Auto2DE, we analyzed data sets from 52 healthy mouse cine loops and 14 healthy rat cine loops (validation cohorts) with both software tools and compared average values for cardiac function parameters in spider plots (Fig. 1d, e), which revealed excellent agreement between 2DE and conventional Auto2DE in mice (Fig. 1d). LV function patterns of rat cine loops shared main characteristics, however, absolute values differed, especially when focusing on absolute end-systolic volume (ESV) and end-diastolic volume (EDV) and the resulting relative measure EF. A post-hoc power analysis regarding both validation cohorts can be found in the Additional file 1.

### The accuracy of Auto2DE-assessed data is dependent on cine loop quality

**Fig. 2:**
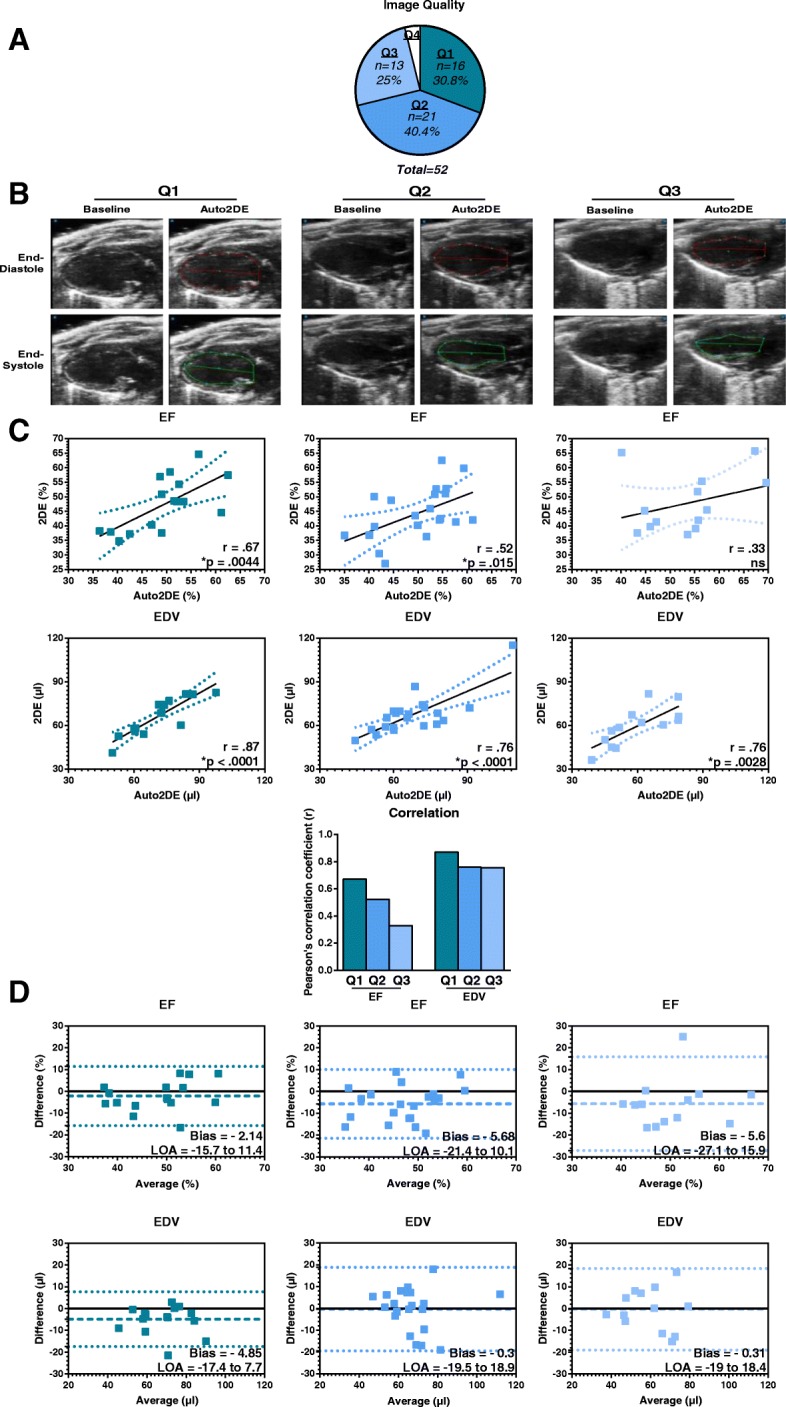
Analysis of murine cine loop data with 2DE and Auto2DE stratified by image quality. **a** Color-coded distribution of cine loops with distinct image quality (Q1-Q4) in a cohort of 52 mouse cine loops. **b** Exemplary cine loops +/− tracings by Auto2DE stratified by image qualities (Q1-Q3). **c** Pearson’s correlation analysis and **d** Bland-Altman analysis of Q1-images (left panel), Q2-images (middle panel) and Q3-images (right panel) of Auto2DE and 2DE-assessed EF and EDV data. Numbers in brackets indicate n-numbers. Mean + SEM. r: Pearson’s correlation coefficient. LOA: Limits of Agreement. *p < .05 for correlation analysis

To investigate the degree to which image quality affects the performance of Auto2DE analysis, cine loops of the murine validation cohort were graded into four distinct quality levels (Q1 - good, Q2 - fair, Q3 - poor, or Q4 - insufficient) by an expert in small animal echocardiography (Fig. 2a). Two cine loops showed inferior image quality (Q4) due to poor endocardial border delineation or segment visibility and were excluded from further analyses. Exemplary images of quality levels and corresponding manual and automated tracings indicate the increasing challenge of endocardial border tracing with decreasing image quality (Fig. 2b). Next, we correlated values derived from both methods and calculated the correlation coefficient for the stratified data sets (Table 1**,** Fig. 2c). The results were in line with the exemplary tracings in that correlation between manual and automated tracings decreased as a function of image quality. For example, for Q1-stratified cine loops, all LV function parameters showed good to very good correlations between both methods (Table 1). Correlation coefficients decreased in Q2 and even further in Q3 stratified images, indicating that poor image quality caused enhanced variabilities regarding cardiac function analysis between both techniques (Fig. 2c). In contrast, correlation coefficients for EDV remained stable independent of image quality. However, correlations were still best for cine loops of Q1 stratified data.

**Table 1:** Validity of automated tracings is dependent on image quality

		Pearson’s r	95% CI	*p*-value	Equation	Bias	LOA	Significance of Bias
**Q1 (*n*** **= 16)**	**EF (%)**	.67*	.26 to .88	**.0044**	Y = 0.8312x + 6.223	−2.14	−15.7 to 11.4	n.s.
	**FS (%)**	.77*	.44 to .92	**.0005**	Y = 2.082x-15.3	−1.20	−8.9 to 6.4	n.s.
	**EDV (**μl**)**	.87*	.66 to .95	**<.0001**	Y = 0.8465x + 6.08	−4.85	−17.4 to 7.7	n.s.
	**ESV (μl)**	.73*	.38 to .90	**.0012**	Y = 0.8159x + 5.644	−1.05	−15.6 to 13.5	n.s.
	**SV (μl)**	.88*	.68 to .96	**<.0001**	Y = 0.9732x-2.87	−3.81	−10.8 to 3.2	n.s.
	**CO (μl)**	.88*	.68 to .96	**<.0001**	Y = 0.9543x-1.024	−1.71	−4.7 to 1.3	n.s.
	**HR (bpm)**	.99*	.96 to 1.0	**<.0001**	Y = 0.01201x + 7.861	2.73	−9.2 to 14.6	n.s.
**Q2 (*n*** **= 21)**	**EF (%)**	.52*	.12 to .78	**.015**	Y = 0.6419x + 12.26	−5.68	−21.4 to 10.1	n.s.
	**FS (%)**	.24	−.22 to .61	.2977	Y = 0.2597x + 7.213	−2.21	−9.2 to 4.8	n.s.
	**EDV (μl)**	.76*	.49 to .90	**<.0001**	Y = 0.7203x + 18.6	−0.30	−19.5 to 18.9	n.s.
	**ESV (μl)**	.60*	.23 to .82	**.0039**	Y = 0.6607x + 14.98	3.61	−10.9 to 18.1	n.s.
	**SV (μl)**	.74*	.45 to .88	**.0001**	Y = 0.7098x + 5.966	−3.91	−17.0 to 9.6	n.s.
	**CO (μl)**	.77*	.50 to .90	**<.0001**	Y = 0.7843x + 1.646	−1.64	−7.4 to 4.1	n.s.
	**HR (bpm)**	.92*	.80 to .97	**<.0001**	Y = 0.04046x-20.08	−1.91	−27.8 to 23.9	n.s.
**Q3 (*n*** **= 13)**	**EF (%)**	.33	−.27 to .75	.2726	Y = 0.3706x + 27.93	−5.60	−27.1 to 15.9	n.s.
	**FS (%)**	.56*	.02 to .85	**.0452**	Y = 0.9746x-2.26	−2.60	−12.9 to 7.7	n.s.
	**EDV (μl)**	.76*	.35 to .92	**.0028**	Y = 0.7182x + 16.49	−0.31	−19.0 to 18.4	n.s.
	**ESV (μl)**	.64*	.15 to .88	**.0175**	Y = 0.734x + 10.66	3.13	−14.1 to 20.4	n.s.
	**SV (μl)**	.27	−.33 to .72	.3687	Y = 0.2637x + 19.62	−3.44	−19.7 to 12.8	n.s.
	**CO (μl)**	.27	−.33 to .71	.3757	Y = 0.2304x + 9.395	−1.80	−8.5 to 4.9	n.s.
	**HR (bpm)**	.92*	.76 to .98	**<.0001**	Y = -0.08216x + 43	4.76	−26.0 to 35.5	n.s.

*ESV* End-Systolic Volume, *EDV* End-diastolic Volume, *SV* Stroke Volume, *EF* Ejection Fraction, *FS* Fractional Shortening, *CO* Cardiac Output, *HR* Heart Rate, *LOA* Limits of Agreement. *Data in bold are statistically significant

As a next step, we displayed data from both analysis tools as Bland-Altman plots to calculate bias and LOA (Limits of Agreement) (Table 1**,** Fig. 2d). As compared to mean absolute values (Fig. 1d) bias levels were small and largely independent from image quality, indicating the absence of systematic errors. LOA levels, however, increased with poorer image quality. This is again exemplary shown for the clinically important metrics EF and EDV (Fig. 2d).

### Auto2DE performance is accurate in severe pathologic cardiac phenotypes

To test whether automated tracing is suitable to detect pathologic alterations in cardiac performance, we applied Auto2DE in small animal models of distinct cardiac pathologies. Physiological validation of the phenotype of individual models including mean values of echocardiographic data are shown in Table 2, and Additional file 1: Tables S3-S4, but will not be extensively discussed here.

**Table 2:** Phenotypic characteristics of pathophysiological small animal models

Species	Modell	Strain	Age (wks)	HW (mg)	BW (g)	HW/ BW-ratio	Blood Glucose (mg/dl)	Other
**Mouse**	**Ctrl**	*Ncor1*^*tm1Anh*^/J	20	150.5 ± 10.1 (*n* = 4)	33.1 ± 2.3	4.46 ± 0.1 (*n* = 4)	163 ± 15	–
	**Type I DM**	*Ncor1*^*tm1Anh*^/J	20	141.5 ± 17.7 (*n* = 2)	28.3 ± 2.6	5.34 ± 0.2 (*n* = 2)	537 ± 101	–
	**Ctrl**	db/db+	20	158.2 ± 2.6	32.2 ± 0.6	4.92 ± 0.1	146 ± 23.9	–
	**Type II DM**	db+/db+	20	124.8 ± 1.9	30.4 ± 1.7	3.88 ± 0.2	514 ± 68.6	–
	**Ctrl**	129/Sv	8–10	113.7 ± 2.8^a^	26.8 ± 1.7^a^	4.24 ± 0.1^a^	156 ± 8^a^	–
	**Iso**	129/Sv	8–10	109.3 ± 2.6^a^	26.6 ± 1.8^a^	4.11 ± 0.1^a^	145 ± 6^a^	–
	**Ctrl**	C57BL/6J	18–19	123.0 ± 8.3	28.0 ± 0.3	4.40 ± 0.1	192.7	Gradient P^b^
							±24.7	−2.17 ± 1.1
	**TAC**	C57BL/6J	18–19	160.3 ± 29.1	28.9 ± 0.3	5.72 ± 0.3	192.2	Gradient P^b^
							±40.6	32.22 ± 3.2
**Rats**	**Ctrl**	tetO-shIR	18	–	427 ± 6.8	–	108.5 ± 1.5	–
	**TetO**	tetO-shIR	26	–	404 ± 5.3	–	427.5 ± 23	–
	**mRen**	mRen27	26	–	438 ± 12.7	–	117 ± 2.5	–
	**TetO/mRen**	mRen27/tetO-shIR	26	–	345 ± 12.9	–	309 ± 25.1	–
	**Ctrl**	Sprague Dawley	7	–	180 ± 7.4	–	–	–
	**dTGR**	female:TGR(hRen)L10 J	7	–	170 ± 2.8	–	–	–
		male:TGR(hAogen)L162 3						

*HW* Heart weight, *BW* Body weight, *HW/BW-ratio* heart weight/Bodyweight-ratio. ^a^Data published previously in [13]. ^b^Data published previously in [7]. Gradient P assessing the degree of aortic stenosis was calculated from velocity parameters 10 weeks post-TAC as described previously [30, 31]. *Data in bold are statistically significant

**Fig. 3:**
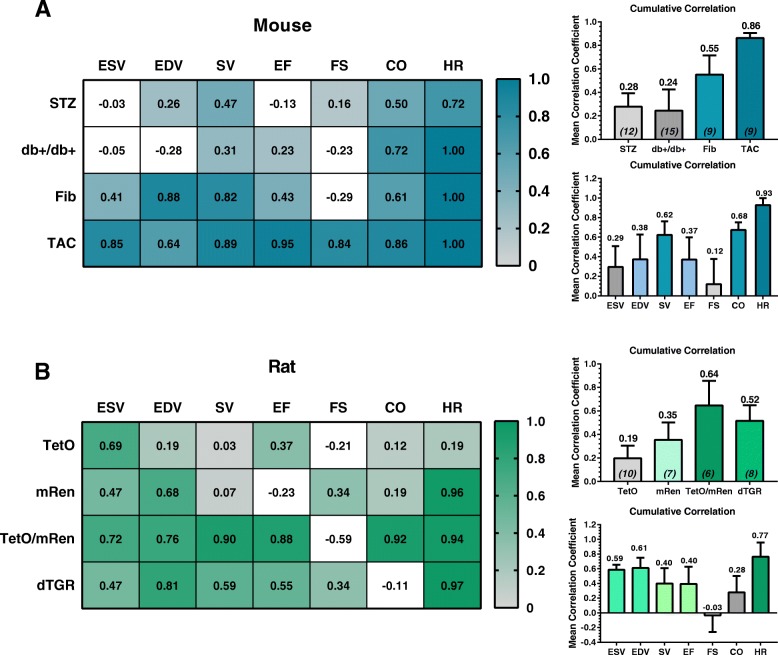
Correlation analysis of 2DE and Auto2DE-assessed data in cardio-pathophysiological conditions. **a** Color-coded heat map of correlation analysis between the two methods of interest in four mouse models and **b** four rat models with cardiac phenotypes. Bar graphs next to the heat maps indicate the averaged correlation coefficient *r* for animal models or cardiac function parameters, demonstrating applicability of Auto2DE to analyze an individual animal model or cardiac function parameter. Bold-printed numbers indicate mean *r*-values of correlation analysis. Numbers in brackets indicate n-numbers. Mean + SEM

We investigated four mouse models (STZ: Type I DM, db+/db+: Type II DM, Fib: subendocardial fibrosis, TAC: LV failure) and four rat models (TetO: genetically-induced DM, mRen: hypertension, TetO/mRen: metabolic syndrome, dTGR: HFpEF) with 2DE and novel Auto2DE and correlated the obtained data sets. Color-coded heat maps of correlation coefficients and corresponding bar graphs of averaged correlation coefficient from all seven cardiac function parameters (ESV, EDV, SV (stroke volume), EF, FS (fractional shortening), CO (cardiac output), HR (heart rate)) or from all four animal models, respectively, reflect the suitability of Auto2DE in individual animal models or with respect to measurement of individual cardiac parameters, respectively (Fig. 3a, b).

Surprisingly, similar patterns of correlations emerged for individual animal models in both species, in that diabetic models (mice: STZ, db^+^/db^+^; rats: TetO) yielded the poorest cumulative correlation. In contrast and against our original hypothesis that strong phenotypes would be less suitable for an automated analysis algorithm, in both species models with pronounced cardiac phenotypes and markedly impaired LV function showed good to very good correlations (TAC: r = .86; TetO/mRen: r = .64) (Fig. 3a, b). Individual LV function parameters also differed with respect to their accurate assessment by Auto2DE. Heart rate was by far the parameter with best agreement between methods in both species (mice: r = .93; rats: r = .77). In mice the relative metrics SV and CO correlated well between software tools. In rats, analysis of LV dimensions yielded good cumulative correlations for the absolute volumes ESV and EDV, while correlation was poor for relative metrics SV and EF.

**Fig. 4:**
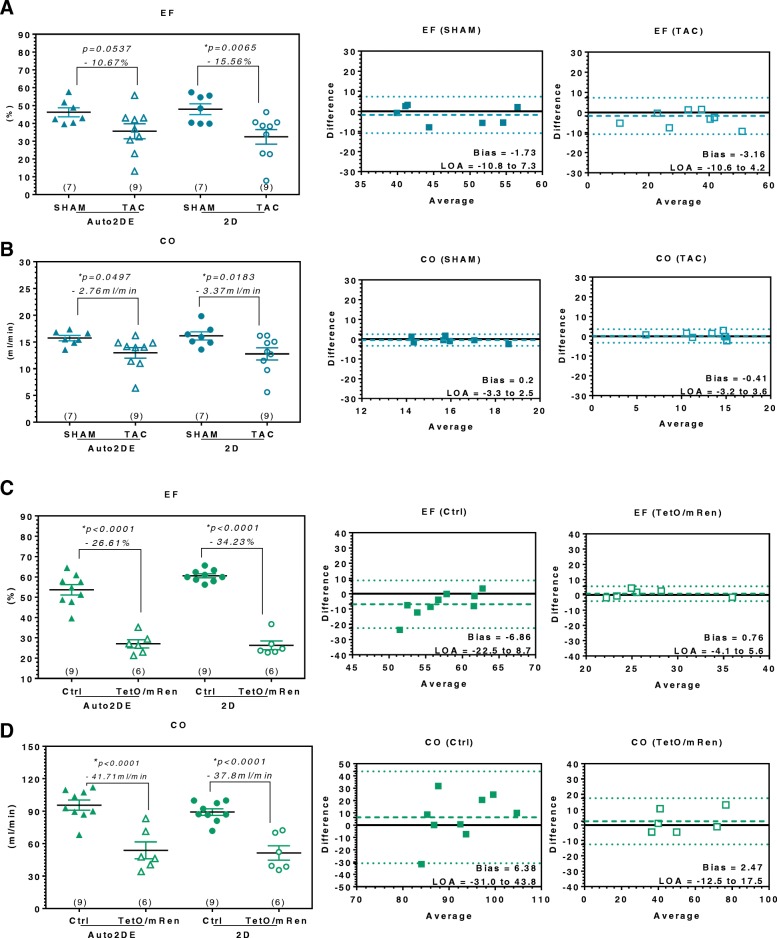
Method comparison of Auto2DE and 2DE in pathologies with pronounced alterations of LV function. **a** Mean EF- and **b** CO-difference between SHAM-mice and TAC-mice assessed with novel Auto2DE and 2DE. Bland-Altman analysis was stratified by healthy and diseased mice. **c** Mean EF- and **d** CO-difference between Ctrl-rats and TetO/mRen-rats assessed with novel Auto2DE and 2DE. Bland-Altman analysis was stratified by healthy and diseased rats. Numbers in brackets indicate the n-numbers. LOA: Limits of Agreement. *p < .05 vs. corresponding control-group analyzed with the same imaging technique

A closer look at the method comparison in pronounced cardiac pathologies revealed that Auto2DE is in general suitable to detect strong phenotypes, defined as impaired LV function in terms of reduced EF accompanied by significant cardiac remodeling (e.g. endocardial fibrosis, inflammation, hypertrophy). However, when directly comparing TAC-mice to their corresponding SHAM-controls, Auto2DE failed to detect significant reduced EF, while conventional 2DE reliably detected the expected decreased EF (Fig. 4a). The concomitant decrease in the clinically relevant parameter CO in TAC-mice as compared to SHAM-controls was detected with statistical significance by both techniques, indicating the general capacity of Auto2DE for detection of impaired cardiac performance (Fig. 4b). Bland-Altman analysis of TAC mice and SHAM-controls showed very good agreement and minimal bias between automated and semi-automated software tools and no differences in the analysis of healthy or diseased mice (Fig. 4b, c). These results were corroborated by data from TetO/mRen-rats as a model of metabolic syndrome (Fig. 4c, d). Both Auto2DE and conventional 2DE detected a highly significant reduced EF and CO in diseased as compared to control rats (Fig. 4c).

### Poor image quality is not the cause for poor performance of Auto2DE in diabetic animal models

**Fig. 5:**
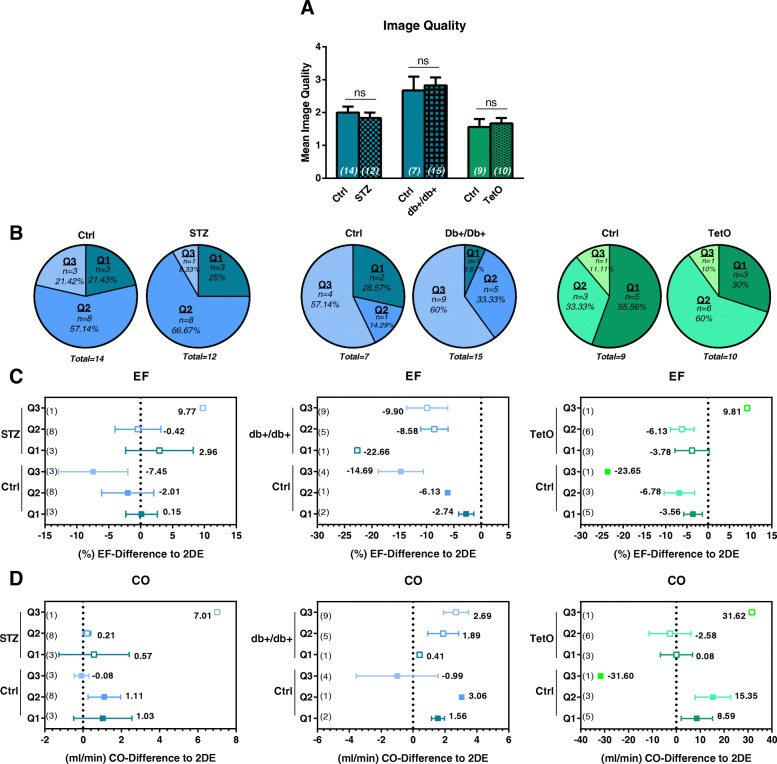
Method comparison of Auto2DE and 2DE of cine loops stratified by image quality in diabetic animal models. **a** Mean image quality of diabetic cohorts and their corresponding study-controls. **b** Distribution of image quality levels in healthy and diabetic mice and rats. **c** Mean difference between Auto2DE and 2DE absolute EF and **d** CO values in diabetic animals and corresponding controls. The data was stratified by image quality levels prior to the calculation of mean differences. Bold-printed numbers indicate mean difference between the techniques. Numbers in brackets indicate n-numbers. Mean + SEM

In contrast to our original hypothesis that pronounced cardiac phenotypes would in general be less suitable for automated analysis tools, we found Auto2DE analysis to be specifically hampered in three diabetic animal models. Based on the impact of image quality on Auto2DE performance shown previously in this study, we speculated that diabetic cardiomyopathy may result in poor image quality per se. To test this notion, we compared mean image quality of diabetic animals and their corresponding healthy controls of the same study (Fig. 5a). While individual studies differed in averaged image quality, no differences in image quality were detected between healthy and diabetic animals, indicating that diabetic conditions did not worsen image quality per se (Fig. 5a, b). Next, we calculated the mean difference between Auto2DE and 2DE for the parameters EF and CO for healthy and diseased animals stratified by image quality, to probe whether Auto2DE is similarly image quality-dependent in diabetic conditions as previously shown for healthy animals (vide supra) (Fig. 5 c, d). Mean differences and standard deviations between Auto2DE and 2DE increased as a function of quality level in healthy controls, corroborating the results from the murine validation cohort. A similar pattern was observed in diabetic animals, suggesting that poor image quality had similar effects on the performance of Auto2DE in both healthy and diseased animals. Taken together, these findings exclude poor image quality as the predominant cause for the poor performance of Auto2DE in diabetic animal models.

## Discussion

In the present study, we evaluated a novel automatic software tool for LV function assessment against the manual 2DE standard of use method. Our results revealed that (1) data analysis with Auto2DE is rapid, straightforward and independent of observer experience levels, (2) Auto2DE-assessed data correlated with manually assessed 2DE data with negligible mean differences or bias and within reasonable LOAs in healthy mice, yet less so in rats, (3) correlations between both techniques were dependent on image quality, indicating that Auto2DE performance decreases as a function of image quality, (4) pronounced cardiac phenotypes showed in general good agreement between methods, with the notable exception of diabetic animal models which seem less suitable for analysis with Auto2DE.

A major advantage of novel Auto2DE is the independence of observer experience level and hence, a small intra- and interobserver variability, which became evident when tracing the same cardiac cycle twice with Auto2DE, beginning with the same frame, always leads to the same data set (data not shown). In contrast, observer variabilities and operator experience levels are known as major drawback of manually analyzed echocardiographic data sets [19–21]. The requirement for extensive training, and the remaining time-consuming task of manual tracing have fuelled interest within the basic research community into automated software approaches for the assessment of echocardiographic data that can rapidly generate data sets with high reproducibility and independent of observer experience. However, there seem to be differences regarding the tracing with Auto2DE between mice and rats, since absolute values of Auto2DE-assessed LV function parameters differed more in rats compared to mice. Due to the rat’s larger size, physiological noise originating from cardiac and respiratory motion is larger as compared to mice. As cardiorespiratory noise is well known to cause severe artifacts, such an effect may hamper the proper analysis of rat cine loops by Auto2DE [22–24]. Another explanation for this finding could be an underrepresentation of rats among the 200 expertly curated LV analysis traces used to train the Auto2DE algorithm (which is unknown as the original tracing library is not public domain).

Yet, automated analyses may be critically hampered by observer-independent factors such as poor image quality; however, this problem applies equally to conventional analyses. Previous studies have highlighted the impact of image quality on the reliability of the produced data for both manually assessed 2DE [25] or 3DE [26] data sets. It is thus little surprising that also in automated approaches image quality influences analytical performance. The relevance of this finding should be emphasized, as image quality of the data set thus emerges as a pivotal factor contributing to future decision making regarding the choice of analysis tools utilized in small animal studies. In the present study and in line with clinical automated software approaches, we decided to include all cine loops with adequate quality for Auto2DE and 2DE and performed no further preselection based on image quality in order to realistically mimic experimental routines of animal studies [10, 11]. Future studies may consider possible exclusion criteria for cine loops with poor image quality to increase reliability of Auto2DE when utilized in murine studies.

We originally hypothesized that phenotypes with severely altered cardiac function may not be suitable for Auto2DE analysis, as such pathologies may be associated with poor image quality. Conversely, we observed that pronounced cardiac phenotypes, namely a mouse model of LV failure and a rat model of metabolic syndrome, were suitable for the analysis with Auto2DE and showed the best correlation with 2DE-derived data of all tested animal models. In contrast, subtle alterations of cardiac phenotypes, caused by DM, fibrosis or hypertension showed less convincing results, when assessed with Auto2DE. Along similar lines, Auto2DE was not able to show the expected significant reduction of EF in TAC-mice as compared to SHAM-controls, pointing towards a lower sensitivity of novel Auto2DE compared to manual 2DE. The observed results underline the importance of sensitivity in diagnostic imaging technologies with echocardiographic results building the basis for clinical decision-making and research interpretation, respectively. Notably, none of the clinical approaches with automated 2DE software tools have yet addressed the question whether automated software tools may be less or more suitable for individual pathologies despite their use in individual clinical studies [11, 12, 27]. We conclude from our data that the current version of the Auto2DE software tool is sufficiently sensitive for quantitative diagnosis of pronounced cardiac pathologies and associated severe alterations in cardiac function, while its usefulness for the analysis of early cardiac damage or subclinical disease stages such as seen in diabetic animal models is still limited.

Clinical approaches of automated software tools demonstrated very good agreement rates for the clinically relevant parameter EF, with partially similar or better accuracy as established control methods [9, 11, 12]. In the present study, we can only speculate as to the reasons for the poor agreement rates of EDV and ESV in mice. One confounding factor might be the choice of two murine diabetic models, namely STZ and db^+^/db^+^, which demonstrated poor outcome in correlation analysis of all cardiac function parameters. When excluding diabetic mouse models (just Fib and TAC) from the cumulative correlation analysis, ESV and EDV parameters reach good correlations, which are comparable to SV and EF calculated correlations. This murine effect was replicated in a diabetic rat model (TetO) which again showed the worst outcome in correlation analysis of all included animal models. As such, our data suggest that the safe and valid use of automated software tools is specifically hampered in diabetic disease conditions. Notably, both type I and II DM cause pathologic heart rate variabilities in mice [28, 29]. In line with this notion, Stables and colleagues reported previously a relative reduction of sympathetic control of HR for type I DM STZ model and an altered circadian rhythm of sympathetic HR-control for db^+^/db^+^-mice [29]. Notably, our own data yield a poor correlation of HR measured with Auto2DE as compared to manually assessed HR in STZ and TetO animal models, yet not in animal models of other cardiac pathologies. HRs measured via an electrophysiological signal, i.e. R-wave (end-diastole) and P-wave (end-systole), are the basis for the novel Auto2DE technique, which automatically searches for the end-systolic frame based on the electrocardiography (ECG)-signal. In contrast, experienced observers often visualize primarily the maximum and minimum dimension of the LV in the B-mode cine loop and use the ECG-signal only in a secondary manner for the manual analysis of LV function. Analysis of B-mode images by the automated software tool or manual tracing of the observer is therefore based on different parameters (ECG-signal vs. B-mode image). When we compared the amount of frames analyzed by automated and manual tracings, we realized that the number of analyzed frames sometimes differed between both techniques (data not shown), possibly leading to poor outcome of Auto2DE in diabetic animal models. Moreover, the automated algorithm was probably originally not trained with cine loops from diabetic animals, therefore hampering the analysis of the same (information was not accessible from the company). Even if we cannot prove this hypothesis with the available data sets, a relationship of the type of cardiac pathology and suitability for the analysis with Auto2DE seems to exist.

Our study has some limitations, which should be taken into account when interpreting the presented data set. First, mouse and rat models analyzed with 2DE and novel Auto2DE were of different age. The possibility that aging as a pathophysiological process itself could have had impact on cardiac performance was not investigated and cannot be ruled out in our methodological approach to compare both aforementioned imaging modalities. Future studies may address whether Auto2DE is suitable for the analysis of age-associated cardiac function decline. Second, while animals were positioned on a 37 °C heatpad during image acquisition we did not monitor body temperature directly. Therefore, we cannot fully exclude that variations in body temperature had potential confounding effects on the assessment of cardiac performance in the present study. In the present study, we opted to use the MX400 linear array transducer due to its superior performance in tissue penetration for the echocardiographic examination of mice cohorts, which however comes at the cost of a slightly lower spatial resolution as compared to the MX550D linear array transducer (Visualsonics). Furthermore, post-hoc power analysis demonstrated that the sample size in the rat validation cohort was not sufficient to detect differences between 2DE and Auto2DE for the relative metrics EF and FS. Ongoing studies using larger sample sizes will thus be required to verify that the tested echocardiographic modalities yield similar results in rats. Lastly, it should be emphasized that correlation analyses reflect relationships rather than agreement between two imaging modalities. The latter was exemplarily addressed in detail by Bland & Altman analyses for two cardiovascular disease models and two cardiac function parameters.

## Conclusion

Fully automated assessment of LV function in small animals by Auto2DE is feasible, fast, and provides precise results comparable to manually assessed data in healthy mice and, albeit to a lesser degree, in rats. Auto2DE sensitively diagnoses severe cardiac pathologies with pronounced alterations of LV function in small animals. However, automated analysis by Auto2DE is hampered by poor image quality and in pathologies with subtle altered LV function such as diabetic cardiomyopathies.

## Additional file

Additional file 1: Online Supplement. (DOCX 511 kb)

## Abbreviations

2DE: Two-dimensional echocardiography
3DE: Three-dimensional echocardiography
Auto2DE: Automated 2D-border detection algorithm
BW: Body weight
CMR: Cardiac magnetic resonance imaging
ECG: Electrocardiography
EDV: End-diastolic volume
EF: Ejection fraction
ESV: End-systolic volume
fps: Frames per second
FS: Fractional shortening
HR: Heart rate
HW: Heart weight
LOA: Limits of agreement
LS: Global longitudinal peak strain
LV: Left-ventricular or left ventricle
SEM: Standard error of mean
SV: Stroke volume
TAC: Transverse aortic constriction

## References

[1] Ram R, Mickelsen DM, Theodoropoulos C, Blaxall BC (2011). New approaches in small animal echocardiography: imaging the sounds of silence. Am J Physiol Heart Circ Physiol.

[2] Szulik M, Pappas CJ, Jurcut R, Magro M, Peeters E, Goetschalckx K (2011). Clinical validation of a novel speckle-tracking–based ejection fraction assessment method. J Am Soc Echocardiogr.

[3] Lang RM, Badano LP, Mor-Avi V, Afilalo J, Armstrong A, Ernande L (2015). Recommendations for cardiac chamber quantification by echocardiography in adults: an update from the American Society of Echocardiography and the European Association of Cardiovascular Imaging. J Am Soc Echocardiogr.

[4] Heinen A, Raupach A, Behmenburg F, Hölscher N, Flögel U, Kelm M (2018). Echocardiographic analysis of cardiac function after infarction in mice: validation of single-plane long-Axis view measurements and the bi-plane Simpson method. Ultrasound Med Biol.

[5] Ponikowski P, Voors AA, Anker SD, Bueno H, Cleland JGF, Coats AJS (2016). 2016 ESC guidelines for the diagnosis and treatment of acute and chronic heart failure: the task force for the diagnosis and treatment of acute and chronic heart failure of the European Society of Cardiology (ESC)developed with the special contribution of the heart failure association (HFA) of the ESC. Eur Heart J.

[6] Dorosz JL, Lezotte DC, Weitzenkamp DA, Allen LA, Salcedo EE (2012). Performance of 3-dimensional echocardiography in measuring left ventricular volumes and ejection fraction. J Am Coll Cardiol.

[7] Grune J, Blumrich A, Brix S, Jeuthe S, Drescher C, Grune T (2018). Evaluation of a commercial multi-dimensional echocardiography technique for ventricular volumetry in small animals. Cardiovasc Ultrasound.

[8] Hoffmann R, Barletta G, von Bardeleben S, Vanoverschelde JL, Kasprzak J, Greis C (2014). Analysis of left ventricular volumes and function: a multicenter comparison of cardiac magnetic resonance imaging, cine Ventriculography, and unenhanced and contrast-enhanced two-dimensional and three-dimensional echocardiography. J Am Soc Echocardiogr.

[9] Muraru D, Badano LP, Piccoli G, Gianfagna P, Del Mestre L, Ermacora D (2010). Validation of a novel automated border-detection algorithm for rapid and accurate quantitation of left ventricular volumes based on three-dimensional echocardiography. Eur Heart J Cardiovasc Imaging.

[10] Barbosa D, Heyde B, Dietenbeck T, Houle H, Friboulet D, Bernard O (2013). Quantification of left ventricular volume and global function using a fast automated segmentation tool: validation in a clinical setting. Int J Cardiovasc Imaging.

[11] Cannesson M, Tanabe M, Suffoletto MS, McNamara DM, Madan S, Lacomis JM (2007). A novel two-dimensional echocardiographic image analysis system using artificial intelligence-learned pattern recognition for rapid automated ejection fraction. J Am Coll Cardiol.

[12] Knackstedt C, Bekkers SCAM, Schummers G, Schreckenberg M, Muraru D, Badano LP (2015). Fully automated versus standard tracking of left ventricular ejection fraction and longitudinal strain. J Am Coll Cardiol.

[13] Beyhoff N, Brix S, Betz IR, Klopfleisch R, Foryst-Ludwig A, Krannich A (2017). Application of speckle-tracking echocardiography in an experimental model of isolated subendocardial damage. J Am Soc Echocardiogr.

[14] Wilck N, Markó L, Balogh A, Kräker K, Herse F, Bartolomaeus H, et al. Nitric oxide–sensitive guanylyl cyclase stimulation improves experimental heart failure with preserved ejection fraction. JCI Insight 2018 [cited 2018 Aug 9];3. Available from: https://insight.jci.org/articles/view/96006. 10.1172/jci.insight.96006 PMC5916255 29467337

[15] Grune J, Beyhoff N, Smeir E, Chudek R, Blumrich A, Ban Z (2018). Selective mineralocorticoid receptor cofactor modulation as molecular basis for Finerenone’s Antifibrotic ActivityNovelty and significance. Hypertension.

[16] Grune J, Benz V, Brix S, Salatzki J, Blumrich A, Höft B (2016). Steroidal and nonsteroidal mineralocorticoid receptor antagonists cause differential cardiac gene expression in pressure overload-induced cardiac hypertrophy. J Cardiovasc Pharmacol.

[17] Kotnik K, Popova E, Todiras M, Mori MA, Alenina N, Seibler J, et al. Inducible transgenic rat model for diabetes mellitus based on shRNA-mediated gene knockdown. Joly E, editor. PLoS One 2009;4:e5124. 10.1371/journal.pone.0005124 PMC2659743 19340286

[18] Langheinrich M, Lee MA, Böhm M, Pinto YM, Ganten D, Paul M (1996). The hypertensive Ren-2 transgenic rat TGR (mREN2)27 in hypertension research. Characteristics and functional aspects. Am J Hypertens.

[19] Johnson TV, Symanski JD, Patel SR, Rose GA (2011). Improvement in the assessment of diastolic function in a clinical echocardiography laboratory following implementation of a quality improvement initiative. J Am Soc Echocardiogr.

[20] Zhang Q, Liang Y-J, Zhang Q-H, Li R-J, Chua Y, Xie J-M (2012). Impact of a dedicated training program on the reproducibility of systolic Dyssynchrony measures using tissue Doppler imaging. J Am Soc Echocardiogr.

[21] McGowan JH, Cleland JG (2003). Reliability of reporting left ventricular systolic function by echocardiography: a systematic review of 3 methods. Am Heart J.

[22] Brau ACS, Hedlund LW, Johnson GA (2004). Cine magnetic resonance microscopy of the rat heart using cardiorespiratory-synchronous projection reconstruction. J Magn Reson Imaging.

[23] Brinegar C, Y-JL W, Foley LM, Hitchens TK, Ye Q, Ho C (2008). Real-time cardiac MRI without triggering, gating, or breath holding. Conf Proc IEEE Eng Med Biol Soc.

[24] Pais-Roldán P, Biswal B, Scheffler K, Yu X (2018). Identifying respiration-related aliasing artifacts in the rodent resting-state fMRI. Front Neurosci.

[25] Nagata Y, Kado Y, Onoue T, Otani K, Nakazono A, Otsuji Y (2018). Impact of image quality on reliability of the measurements of left ventricular systolic function and global longitudinal strain in 2D echocardiography. Echo Res Pract.

[26] Tighe DA, Rosetti M, Vinch CS, Chandok D, Muldoon D, Wiggin B (2007). Influence of image quality on the accuracy of real time three-dimensional echocardiography to measure left ventricular volumes in unselected patients: a comparison with gated-SPECT imaging. Echocardiography..

[27] Maret E, Brudin L, Lindstrom L, Nylander E, Ohlsson JL, Engvall JE. Computer-assisted determination of left ventricular endocardial borders reduces variability in the echocardiographic assessment of ejection fraction. Cardiovasc Ultrasound 2008 [cited 2018 Aug 11];6. Available from: http://cardiovascularultrasound.biomedcentral.com/articles/10.1186/1476-7120-6-55. 10.1186/1476-7120-6-55 PMC2596088 19014461

[28] Arroyo-Carmona RE, López-Serrano AL, Albarado-Ibañez A, Mendoza-Lucero FMF, Medel-Cajica D, López-Mayorga RM (2016). Heart rate variability as early biomarker for the evaluation of diabetes mellitus Progress. J Diabetes Res.

[29] Stables CL, Auerbach DS, Whitesall SE, D’Alecy LG, Feldman EL (2016). Differential impact of type-1 and type-2 diabetes on control of heart rate in mice. Auton Neurosci.

[30] Garcia-Menendez L, Karamanlidis G, Kolwicz S, Tian R (2013). Substrain specific response to cardiac pressure overload in C57BL/6 mice. Am J Physiol Heart Circ Physiol.

[31] Zhao M, Fajardo G, Urashima T, Spin JM, Poorfarahani S, Rajagopalan V (2011). Cardiac pressure overload hypertrophy is differentially regulated by -adrenergic receptor subtypes. Am J Physiol Heart Circ Physiol.

