# Supplementary material for: Accurate assessment of LV function using the first automated 2D-border detection algorithm for small animals - evaluation and application to models of LV dysfunction
Source: Cardiovasc Ultrasound. 2019 Apr 22;17:7. doi: 10.1186/s12947-019-0156-0 (PMC6477743; doi:10.1186/s12947-019-0156-0)
Supplement: Supplementary file 1 — Online Supplement. (DOCX 511 kb) [file 12947_2019_156_MOESM1_ESM.docx]

**ADDITIONAL FILE 1**

**Accurate assessment of LV function using the first automated 2D-border detection algorithm for small animals is image-quality dependent**

Jana Grune, Daniel Ritter, Kristin Kräker, Kathleen Pappritz, Niklas Beyhoff , Till Schütte, Christiane Ott, Cathleen John, Sophie van Linthout, Michael Schupp, Ralf Dechend, Dominic Mueller, Nadine Haase, Tilman Grune, Ullrich Kintscher, Wolfgang M. Kuebler

**Expanded Methods and Results**

*Assessment of tracing time*

Tracing time was measured by three independent observers for mice and rats, respectively, and was defined as the time period required to produce a LV function measurement (with Auto2DE or conventional 2DE) of a single cardiac cycle. The observers had distinct levels of training: 1) a trained expert in small animal echocardiography with more than 5 years of training in conventional echocardiographic techniques and advanced imaging techniques and extensive experience with various preclinical cardiovascular animal models (expert); 2) a trained observer, familiar with the conventional 2DE analysis technique, but not in different cardiovascular pathologies and less than 200 tracings in total (trainee); 3) a beginner in 2DE analysis with no experience in endocardial border tracing (beginner). Tracing time was measured for analyses of both healthy and diseased animals, ensuring realistic average tracing times per animal given that pathologic conditions often preclude fast cine loop analysis. Tracing time was measured from the moment when the first speckle of the end-diastolic endocardial border was set until the last speckle in end-systole was manually placed by the observer. The search time for the appropriate end-diastolic frame was not included into the measured tracing time, as it is identical for both assessment techniques.

**Additional Figures and Tables**

**Additional file 1: Figure S1.** Exemplary picrosirius red staining of cardiac cross sections in healthy and diseased mice of the Fib-cohort.


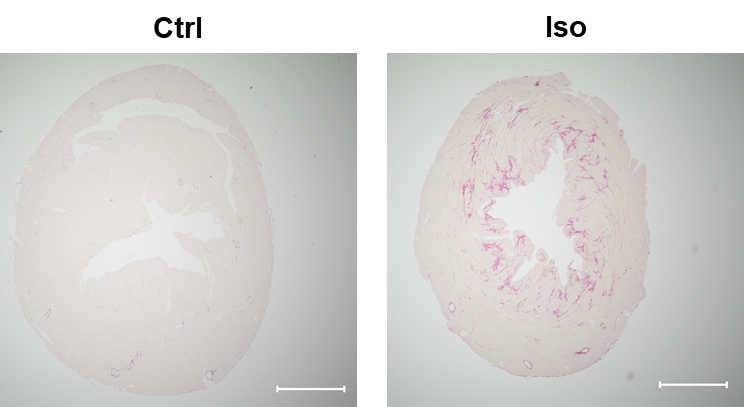


Scale bar: 1 mm.

**Additional file 1: Table S2.** Post-hoc Power Analysis of validation cohorts.

|  | **R²** | **Effect size d** | **α** | **Sample size n** | **Computed power** |
| --- | --- | --- | --- | --- | --- |
| **Mice** |  |  |  |  |  |
| **HR** | 0.8965 | 0.95 | .05  .10 | 52 | **1.00**  **1.00** |
| **ESV** | 0.4603 | 0.68 | .05  .10 | 52 | **0.99**  **0.99** |
| **EDV** | 0.6259 | 0.79 | .05  .10 | 52 | **1.00**  **1.00** |
| **SV** | 0.4762 | 0.69 | .05  .10 | 52 | **0.99**  **1.00** |
| **EF** | 0.2823 | 0.53 | .05  .10 | 52 | **0.99**  **0.99** |
| **FS** | 0.5080 | 0.71 | .05  .10 | 52 | **1.00**  **1.00** |
| **Rats** |  |  |  |  |  |
| **HR** | 0.9987 | 0.99 | .05  .10 | 14 | **1.00**  **1.00** |
| **ESV** | 0.4480 | 0.67 | .05  .10 | 14 | **0.93**  **0.98** |
| **EDV** | 0.8542 | 0.92 | .05  .10 | 14 | **1.00**  **1.00** |
| **SV** | 0.7721 | 0.88 | .05  .10 | 14 | **0.99**  **1.00** |
| **EF** | 0.0339 | 0.18 | .05  .10 | 14 | 0.16  0.27 |
| **FS** | 0.0103 | 0.10 | .05  .10 | 14 | 0.10  0.18 |
| **CO** | 0.6806 | 0.83 | .05  .10 | 14 | **0.99**  **0.99** |

R²: Coefficient of determination, α: type I error

**Additional file 1: Table S3.** Cardiac function analysis of mouse models.

|  | **Auto2DE** | | | | **2DE** | | | |
| --- | --- | --- | --- | --- | --- | --- | --- | --- |
|  | **STZ** | **db+/db+** | **Iso** | **TAC** | **STZ** | **db+/db+** | **Iso** | **TAC** |
| **EF (%)** | 51.7  ±5.0 | 44.7  ±5.9 | 44.4  ±5.8 | 35.6  ±12.5 | 50.4  ±7.4 | 55.1  ±9.2 | 44.8  ±10.7 | 32.4  ±12.2 |
| **FS (%)** | 12.7  ±1.8 | 8.0  ±2.9 | 6.8  ±2.5 | 8.1  ±2.9 | 13.5  ±4.5 | 14.8  ±4.7 | 11.3  ±3.2 | 7.5  ±5.4 |
| **EDV (µl)** | 60.4  ±10.1 | 78.2  ±9.2 | 54.1  ±9.2 | 75.2  ±13.0 | 57.9  ±10.7 | 52.0  ±5.3 | 49.3  ±7.8 | 83.5  ±20.4 |
| **ESV (µl)** | 29.4  ±7.1 | 43.3  ±7.9 | 30.2  ±6.1 | 49.6  ±16.3 | 29.0  ±8.3 | 23.4  ±5.2 | 27.1  ±6.3 | 58.4  ±25.9 |
| **SV (µl)** | 31.0  ±4.8 | 34.9  ±5.2 | 23.9  ±5.3 | 25.6  ±6.9 | 28.9  ±5.2 | 28.6  ±5.6 | 22.2  ±7.0 | 25.2  ±7.6 |
| **CO (mL/min)** | 14.2  ±2.7 | 12.4  ±3.0 | 9.3  ±1.3 | 13.0  ±3.0 | 13.2  ±2.8 | 10.1  ±3.0 | 8.6  ±2.0 | 12.8  ±3.4 |
| **HR (bpm)** | 458  ±42 | 352  ±50 | 397  ±52 | 515  ±51 | 455  ±42 | 350  ±50 | 396  ±51 | 515  ±51 |

Mean + SEM. ESV: End-Systolic Volume, EDV: End-diastolic Volume, SV: Stroke Volume, EF: Ejection Fraction, FS: Fractional Shortening, CO: Cardiac Output. STZ: Streptozotocin, Iso: Isoproterenol, TAC: Transverse Aortic Constriction.

**Additional file 1: Table S4.** Cardiac function analysis of rat models.

|  | **Auto2DE** | | | | | **2DE** | | | |
| --- | --- | --- | --- | --- | --- | --- | --- | --- | --- |
|  | **TetO** | **mRen** | **TetO/mRen** | **dTGR** | **TetO** | | **mRen** | **TetO/mRen** | **dTGR** |
| **EF (%)** | 35.8  ±7.2 | 23.7  ±6.9 | 27.1  ±4.9 | 48.1  ±23.0 | 41.4  ±3.8 | | 38.7  ±4.4 | 26.3  ±5.3 | 67.0  ±7.2 |
| **FS (%)** | 4.6  ±2.6 | 1.6  ±3.3 | 3.3  ±1.6 | 5.0  ±4.5 | 10.9  ±3.3 | | 9.8  ±3.7 | 6.0  ±3.0 | 21.3  ±4.6 |
| **EDV (µl)** | 516.8±68.7 | 889.9  ±119.1 | 588.1±159.8 | 258.8  ±33.9 | 461.5±67.4 | | 765.6  ±102.0 | 599.6  ±218.1 | 209.7  ±59.0 |
| **ESV (µl)** | 332.4±61.8 | 681.9  ±122.8 | 429.1±119.2 | 135.4  ±62.9 | 269.5±36.2 | | 471.1  ±87.6 | 443.4  ±171.7 | 71.8  ±33.8 |
| **SV (µl)** | 184.4±40.1 | 208.1  ±57.3 | 159.0±53.9 | 123.3  ±59.6 | 192.0±38.0 | | 294.5  ±30.4 | 156.2  ±56.0 | 137.9  ±30.7 |
| **CO (mL/min)** | 57.4  ±11.4 | 71.5  ±14.5 | 53.8  ±19.1 | 46.6  ±19.7 | 58.1  ±17.2 | | 98.2  ±15.1 | 51.4  ±16.4 | 55.0  ±10.6 |
| **HR (bpm)** | 313  ±15 | 374  ±165 | 337  ±24 | 409  ±90 | 299  ±52 | | 337  ±71 | 333  ±30 | 407  ±77 |

Mean + SEM. ESV: End-Systolic Volume, EDV: End-diastolic Volume, SV: Stroke Volume, EF: Ejection Fraction, FS: Fractional Shortening, CO: Cardiac Output. STZ: Streptozotocin, Iso: Isoproterenol, TAC: Transverse Aortic Constriction.
